# Performance of SAPS 3 in predicting delirium among critically ill patients

**DOI:** 10.1186/cc10946

**Published:** 2012-03-20

**Authors:** T Cosentino, J Biatto, I Souza, M Dutra, L Ilnicki, P Martins, G Schettino, F Machado

**Affiliations:** 1 Hospital Sírio-Libanês, São Paulo, Brazil

## Introduction

Delirium is a common complication in critically ill patients, occurring in up to 80% of patients on mechanical ventilation [1]. Recent studies showed that sicker patients at ICU admission, assessed by severity scores, are more susceptible to developing delirium [2]. To further evaluate this hypothesis, we undertook this study to assess the performance of SAPS 3 in predicting delirium, among adult patients admitted to a general ICU.

## Methods

This was a prospective observational cohort study performed between June 2010 and June 2011, in a 26-bed ICU at Hospital Sírio-Libanês, São Paulo, Brazil. All consecutive adult patients admitted to the ICU were included. Patients with a previous diagnosis of advanced dementia and those with acute neurological disease (Glasgow <13) were excluded. The evaluation of delirium was performed using the CAM-ICU during routine bedside rounds in the morning. Discrimination and calibration of SAPS 3 in predicting delirium were assessed by the area under the receiver operating curve (AUR ROC) and the goodness of fit (GoF) test, respectively. Secondary outcomes were hospital mortality and lengths of stay among patients with delirium.

## Results

A total of 225 patients were included. The incidence of delirium was 24%. Patients who develop delirium during the ICU stay were older (OR 1.04, 1.02 to 1.07) and more likely to have a previous diagnosis of hypertension (OR 2.36, 1.24 to 4.52). The SAPS 3 (OR 1.09, 1.06 to 1.13) score, SOFA (OR 1.23, 1.09 to 1.39) score, and mechanical ventilation requirement (OR 3.6; 1.35 to 9.60) were higher among patients with delirium. These patients had longer ICU and hospital length of stay, and a higher crude mortality rate (24.07 vs. 7.02%). In a multivariate analysis, age (OR 1.03, 1.00 to 1.05), use of mechanical ventilation (OR 3.91, 1.22 to 12.96) and SAPS 3 score (OR 1.08, 1.04 to 1.12) were independently associated with delirium. SAPS 3 performed well in predicting delirium with an AUR ROC of 0.785 (0.714 to 0.856, best cut-off value ≥54 points) and a GoF of 0.175.

## Conclusion

We found that SAPS 3 was a good parameter for predicting delirium during the ICU stay. Future studies are needed to confirm our results in a larger and different patient sampling.
